# Daily Sampling of an HIV-1 Patient with Slowly Progressing Disease Displays Persistence of Multiple *env* Subpopulations Consistent with Neutrality

**DOI:** 10.1371/journal.pone.0021747

**Published:** 2011-08-02

**Authors:** Helena Skar, Ryan N. Gutenkunst, Karin Wilbe Ramsay, Annette Alaeus, Jan Albert, Thomas Leitner

**Affiliations:** 1 Department of Virology, Swedish Institute for Infectious Disease Control, Solna, Sweden; 2 Department of Microbiology, Tumor and Cell Biology, Karolinska Institute, Stockholm, Sweden; 3 Theoretical Biology and Biophysics, Los Alamos National Laboratory, Los Alamos, New Mexico, United States of America; 4 Department of Molecular and Cellular Biology, University of Arizona, Tucson, Arizona, United States of America; 5 Department of Medicine, Karolinska Institute, Stockholm, Sweden; INSERM, France

## Abstract

The molecular evolution of HIV-1 is characterized by frequent substitutions, indels and recombination events. In addition, a HIV-1 population may adapt through frequency changes of its variants. To reveal such population dynamics we analyzed HIV-1 subpopulation frequencies in an untreated patient with stable, low plasma HIV-1 RNA levels and close to normal CD4+ T-cell levels. The patient was intensively sampled during a 32-day period as well as approximately 1.5 years before and after this period (days −664, 1, 2, 3, 11, 18, 25, 32 and 522). 77 sequences of HIV-1 *env* (approximately 3100 nucleotides) were obtained from plasma by limiting dilution with 7–11 sequences per time point, except day −664. Phylogenetic analysis using maximum likelihood methods showed that the sequences clustered in six distinct subpopulations. We devised a method that took into account the relatively coarse sampling of the population. Data from days 1 through 32 were consistent with constant within-patient subpopulation frequencies. However, over longer time periods, i.e. between days 1…32 and 522, there were significant changes in subpopulation frequencies, which were consistent with evolutionarily neutral fluctuations. We found no clear signal of natural selection within the subpopulations over the study period, but positive selection was evident on the long branches that connected the subpopulations, which corresponds to >3 years as the subpopulations already were established when we started the study. Thus, selective forces may have been involved when the subpopulations were established. Genetic drift within subpopulations caused by *de novo* substitutions could be resolved after approximately one month. Overall, we conclude that subpopulation frequencies within this patient changed significantly over a time period of 1.5 years, but that this does not imply directional or balancing selection. We show that the short-term evolution we study here is likely representative for many patients of slow and normal disease progression.

## Introduction

The HIV-1 envelope gene (*env*) displays the largest genetic diversity in the HIV-1 genome. The evolutionary rate (nucleotide substitution rate) of *env* is affected by the strength of the pressure of the immune system [1], [2] so that both the immune pressure and the evolutionary rate are higher during the chronic, asymptomatic phase than during end-stage disease. Similarly, the immune pressure in long-term non-progressors lasts longer and is often stronger than in typical patients. Thus, HIV-1 genetic evolution in *env* during the chronic disease stage has been characterized by positive selection for escape mutants due to continuous immune surveillance [3], [4], [5], [6]. However, other studies have found HIV-1 evolution during chronic infection to be consistent with a neutral model of evolution, characterized by small effective population sizes (*N_e_*) strongly influenced by random genetic drift [7], [8]. Whether the mutation process will be deterministic or stochastic is generally believed to be dependent on the population size. Deterministic models assume an infinite population size, which given the large amount of HIV-1 particles produced daily in an infected individual (10^10^ virions/day) is not unreasonable [9]. However, it has been proposed that the *N_e_* of HIV-1 during chronic infection is several orders of magnitude lower [7], [10], [11], [12], which would suggest that stochastic processes could influence HIV-1 evolution. To date, a few models have tried to unify the estimated small *N_e_* and the strong positive selection believed to act on HIV during chronic infection. A meta-population model, where a large collection of small subpopulations is subject to frequent migration, extinction, and recolonization, was shown to agree with the low effective population sizes seen in chronic HIV infection [12]. Another example is a combination of both directional and neutral forces acting on the HIV population, where random genetic drift of neutral mutations predominates combined with brief episodes of directional selection [7]. A combination of the two, where the meta-population model and selective sweeps both are factors that act together to reduce the intra-host effective population size of HIV-1 has been proposed to be the most likely explanation of the reduced *N_e_* [13]. Thus, it is still unclear how HIV diversity is affected by selection in an infected individual, and furthermore on which time scale selection operates.

Here we compare short-term (days, weeks, months) and long-term (years) HIV-1 evolution in a treatment naïve, asymptomatic patient with low plasma HIV-1 RNA levels (viral load) and fluctuating, often close to normal CD4+ T-lymphocyte (CD4) counts. In patients like this the immune system generally puts a strong pressure on the virus for a longer time than in typical patients that, in the absence of antiretroviral drugs, develop AIDS quicker. We find that multiple distinct subpopulations persist over years, but that their frequencies fluctuate over time. The fluctuations during the time period of days to months showed no significant signature of variable selection across sequence sites, and the fluctuations were consistent with a neutral model of evolution. Hence, we find no need for balancing selection to explain the persistence of the subpopulations over these time intervals. However, over the period of years, we could detect a signal of positive selection, especially at potential N-linked glycosylation sites (PNGS), which may have shaped the subpopulation structure. Finally, we show that it is important to correctly handle subpopulation fluctuations when using genetic distances to estimate the number of *de novo* mutations.

## Results

### Sequence data

Seventy-seven individual virus sequences of approximately 3100 nucleotides covering *vpu*, *env* and the first half of *nef* were analyzed. The sequences were sampled by limiting dilution from plasma samples obtained at 9 different time points spanning a period of 3 years (Table 1). The limiting dilution sequencing methodology (aka. SGA and SGS) applied here ensures that PCR and sequencing artifacts are virtually absent in the sequences [14], [15]. The majority of sequences were sampled during a time period of 32 days, where the first time point was called day 1. In addition, two samples were drawn approximately 1.5 years before (day −664) and 1.5 years after (day 522) the main sampling period. At each time point 7 to 11 sequences were generated with exception for the earliest time point from which only 3 sequences could be amplified. As this patient was a slow progressor with low virus load it was difficult to obtain additional sequences (see Methods). Only 2 sequences were identical (at day 18), whereas all other sequences were unique.

**Table 1:** Sequence data.

Day	No. clones	RNA load	MPD^a^				Fluctuate			Recombine			
		(copies/ml)	θ^b^(wRec)	*θ* b(noRec)	Ne^d^(wRec)	Ne^d^(noRec)	*θ* b	G^c^	Ne^d^	*θ* b	G^c^	Ne^d^	r^g^
−664	3	n.d.^e^	0.024	n.d.	358	n.d.	n.a.^f^	n.a.	n.a.	n.a.	n.a.	n.a.	n.a.
1	9	832	0.037	0.036	537	531	0.051	58.2	744	0.036	56.0	526	1.34E-07
2	8	794	0.029	0.029	433	433	0.044	42.1	645	0.034	39.4	500	1.49E-02
3	7	1220	0.033	0.033	487	487	0.038	37.9	559	0.038	44.0	565	2.28E-02
11	8	563	0.024	0.024	358	358	0.025	12	362	0.023	2.8	337	2.40E-07
18	10	518	0.014	0.007	204	97	0.015	−24.4	218	0.013	−20.3	190	1.03E-02
25	11	450	0.033	0.033	492	492	0.035	21.8	512	0.036	28.0	526	2.18E-02
32	11	600	0.034	0.033	494	482	0.036	32.9	532	0.041	42.8	608	3.86E-02
522	10	n.d.	0.027	0.021	393	304	0.036	21.8	523	0.036	34.5	526	1.01E-02
Mean:	9.3	711	0.028	0.027	417	398	0.035	25.3	512	0.032	28.4	472	1.48E-02
STD	1.5	264.6	0.007	0.010	103	143	0.011	24.6	162	0.009	25.1	139	1.29E-02

a Mean Pairwise Distance, as measured by PAUP* using a GTR substitution model.

b Genetic diversity (substitutions/site).

c Exponential growth rate.

d Effective population size determined from *θ* = 2N_e_μ with μ = 3.4×10^−5^ substitutions site^−1^ generation^−1^.

e No data.

f Not analyzed because of the small sample size.

g Recombination rate, C/μ, where C is the rate of recombination per inter-site link per generation, and μ is the substitution rate per site per generation.

In total, 18 unique deleterious mutations were observed, including 9 nucleotide substitutions that caused stop codons and 9 that caused frame shifts. Because deleterious mutations are unlikely to survive to the next generation, this suggests a minimum rate of 7.67×10^−5^ deleterious substitutions per site per generation, i.e., in the same order of magnitude as other point mutations and recombination occur [16], [17], [18]. In addition to point mutations, sequence −664.2 had two large deletions, one of 149 nucleotides (nts) in the beginning of gp120 and another of 435 nts in the end of gp41. Sequence 1.10 had two large deletions of 54 and 60 nts in the middle of gp120, and 522.1 had a large deletion of 48 nts in the middle of gp120. In addition, several smaller, mostly in-frame indels, were present, which thus resulted in amino acid insertions or deletions.

### Phylogenetic subdivision

Putative recombinants were identified using the PHI-NNet test. Two sequences (s4.−664.3 and s5.522.10) were classified as putative recombinants within subpopulations and six sequences were identified as putative recombinants with ancestors derived from at least two subpopulations (s2. −664.2, s4. −664.1, s4.18.7, s4.32.9, s4.522.9, s5.1.7) (Figure S1). If these sequences were removed no recombination signal remained in the dataset according to the c-AIC criteria in a GARD Single Breakpoint analysis. To confirm that the identified putative recombinants were robustly identified, we performed 100 iterations of removal of 8 random sequences. None of these iterations rendered the dataset free from recombination signal according to the PHI-NNet test.

**Figure 1:**
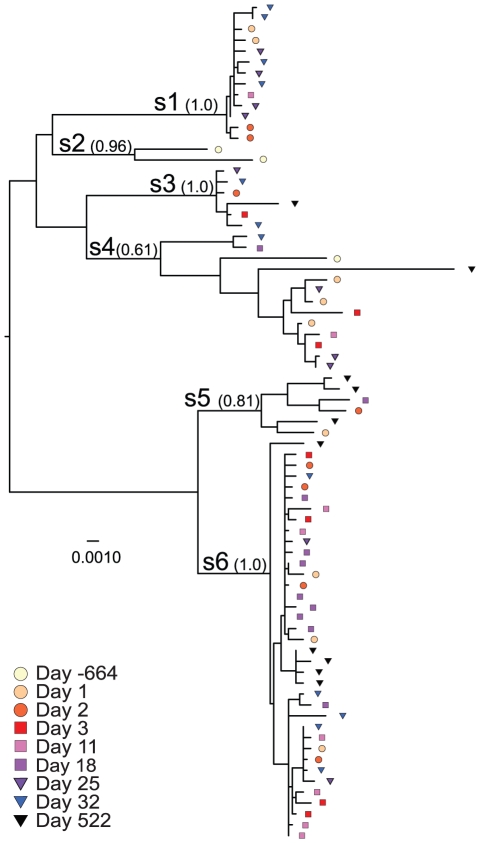
Maximum likelihood tree of the phylogenetic relationships of the viral subpopulations. Sequences from the different time points (in days from day 1) are indicated with different symbols and colors as shown. The subpopulations are labeled with letters s1–s6 and the corresponding bootstrap values are shown as ratios of 1000 replicates.

The general time reversible model with variable rates among sites and a proportion of invariable sites (GTR+G+I) was the best substitution model for our data according to a Modeltest analysis. This model was used to infer a maximum likelihood (ML) tree of the HIV population (Figure 1). The tree displayed six phylogenetic clades, designated as subpopulations s1 through s6, which were all supported by ML bootstrap values 61–100%. Independent of the inferred tree, and thus less affected by any remaining recombination signal, Hudson's population subdivision test supported all subpopulations except s2 at p[K^*^_s_]≤0.0005 (s2 p[K^*^_s_] = 0.0668). A majority of the subpopulations (4 of 5) persisted over the entire study period, if we excluded day −664 that was insufficiently sampled. Thus, at the last time point (day 522), representatives of subpopulations s3, s4, s5 and s6 were still present.

### Subpopulation selection pressure detection is time scale-dependent

To test if the different subpopulations were under different potential selection pressures, we investigated dN/dS ratios on all sites in the sequences and on all branches in the phylogeny. Four dN/dS categories (0, 0.42, 1.24, 10000) were found to best explain the data according to the c-AIC criterion and the GAbranch model available in Hyphy to test lineage specific selection on branches (Figure S2). A majority (79%) of the branches in the tree fell into dN/dS categories 0.42 and 1.24 and of these 72% suggested positive selection, but there was no clear pattern of where in the tree they occurred. The deep branches that connected the subpopulations displayed selection in either direction, i.e., 0.42≤dN/dS≤1.24. Branches displaying either no synonymous or non-synonymous mutations (dN/dS categories 0 and 10000) occurred exclusively within the subpopulations, where the total number of mutations on most of the branches was very low.

In agreement with the branch analyses, codon models could not identify any sites under selection within the subpopulations, suggesting neutral evolution over the time of this study (days 1…522). Furthermore, there was clear evidence of variable selection pressure over sites when we analyzed all subpopulations together in a single phylogenetic tree (p<0.01, likelihood ratio tests with M0 vs. M3: df = 4, and M1a vs. M2a: df = 2). This indicates that individual sites may have been under selection when the subpopulations were established, i.e., over a time much longer than 522 days as the subpopulations already existed and were defined by relatively long branches at day 1 (see further below for an evaluation of how much branches grew over the study period). Interestingly, potential N-linked glycosylation sites (PNGS) were significantly overrepresented among positively selected sites in analyses with the variable selection model M3 (p<0.001, Chi-square test). Further, while both amino acid substitutions and PNGS replacements correlated well with positive selection strength, the response to positive selection was stronger on branches separating the subpopulations than on branches within subpopulations (Figure S3).

Overall, these results suggest that natural selection has little or no impact over short time periods (≤1.5 years), but over long time periods (>3 years) positively selected sites, especially those that involve PNGS, may contribute to the subpopulation structure.

### Subpopulation frequency fluctuations

**Figure 2:**
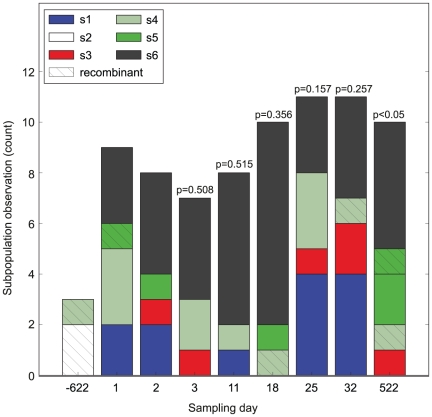
Bar chart showing the observed within-patient frequency fluctuations of the genetic subpopulations during the study period. Subpopulations as defined in Figure 1 are shown in respective colour and recombinant sequences are marked with diagonal stripes. P-values for tests of constant within-patient subpopulation frequencies are shown above the histogram for each day. Thus for each day *J*, subpopulation frequencies *φ_i_* of days 1…*J*-1 are compared to the *φ_i_* frequencies of day *J*. See text for details.

Figure 2 shows the count at which the different subpopulations were observed at each time point. We were interested in understanding the persistence of subpopulations over time. Could these experimental data be expected under a neutral process, or would the data be better explained by selection, and in particular balancing selection?

We first asked whether the experimental data provided evidence that the frequencies of the different subpopulations were changing within the patient. To do so, we used a χ^2^ statistic to assess whether the frequencies observed at each day *J* were consistent with the frequencies observed for the prior days 1…*J-1*. To account for the relatively few samples per time point we accounted for the number of sequences per sample (to limit stochastic sampling effects) and pooled days together when no frequency differences were observed (to increase the power of our analysis). Hence, statistical significance was assessed by simulating both the multinomial sampling of day *J*'s observation, and the inference of the within-patient frequencies for days 1…*J*-1. The resulting p-values for constant within-patient subpopulation frequencies are shown in Figure 2. From this analysis, we observed no statistically significant changes in within-patient subpopulation frequencies over the first 32 days, but at day 522 the frequency fluctuations became statistically significant (p<0.05). As detailed in Supporting Information, repeated analyses that excluded the putative recombinant sequences yielded similar results (Table S1), although the fluctuations at day 522 had a p-value of 0.064.

### Subpopulation frequency fluctuations are consistent with neutral drift

Under a neutral model, the *N_e_* controls the strength of fluctuations in within-patient subpopulation frequencies. Large *N_e_* results in small fluctuations, while small *N_e_* results in large fluctuations. Eventually, if no new subpopulations arise (as was observed in days 1…522), under a neutral model one subpopulation would eventually take over the entire population, eliminating all subpopulation diversity. Thus, we now ask whether the observed diversity at day 522 is consistent with a neutral model, given realistic values for *N_e_*.

Since no significant frequency changes occurred during days 1…32, we pooled those sequences together (n = 64) to derive more accurate subpopulation frequencies (Table 2). Given these inferred frequencies, we compared expected and observed subpopulation frequencies on day 522. We noted a number of potentially unlikely events under a neutral model. Some of these events indicated small frequency fluctuations, and thus large *N_e_*. One was that the observed frequency of subpopulation s6 on day 522 was 5, exactly the expected value. Also, we still observed subpopulations s3 and s4 on day 522, at frequencies similar to that expected from constant within-patient frequencies. Additionally, we observed 4 subpopulations present on day 522, indicating that not much diversity had been lost. Thus, we asked which values of *N_e_* are large enough to be consistent with these observations. On the other hand, other aspects of the data suggested large frequency fluctuations. In particular, subpopulation s1 was not observed on day 522, while our expectation was 2 observations. Hence, we also asked which values of *N_e_* are small enough to be consistent with this observation. Finally, subpopulation s5 was observed at much higher frequency than expected at day 522. For small *N_e_*, we would have expected subpopulation s5 to go extinct, whereas for very large *N_e_* it would have been unlikely to rise to high frequency. Thus we also asked whether any value of *N_e_* makes the observed count of subpopulation s5 plausible. Nearly identical results were observed when putative recombinants were removed (Table S2). Again, these calculations accounted for the stochastic effects of the sampling size at day 522.

**Table 2:** Subpopulation frequencies: inferred, expected, and observed.

Subpopulation	*φ_i_*_,32_±2σ	Expected *f_i,522_* (out of 10)	Observed *f_i,522_*
s1	0.203±0.100	2.03	0
s2	0	0	0
s3	0.078±0.067	0.78	1
s4	0.172±0.094	1.72	1
s5	0.047±0.053	0.47	3
s6	0.500±0.125	5.00	5

**Figure 3:**
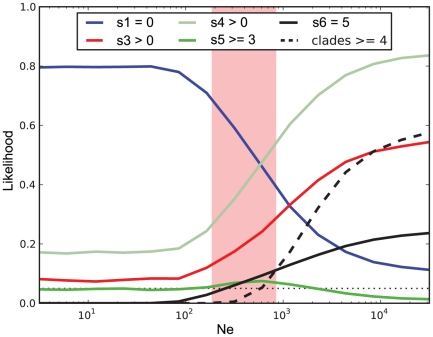
Likelihoods of various aspects of the data under neutral evolution. The pink shaded region denotes the 2σ range of *N_e_* (512±162) inferred using Fluctuate (Table 1) and the dotted line denotes a cut-off at *p* = 0.05.

Independently, the genetic diversity (*θ*) and *N_e_* were estimated using three methods (Table 1). The results were similar and unaffected by the exclusion of putative recombinants. The estimates based on Fluctuate showed that *θ* ranged from 0.015 to 0.051 substitutions/site during the study period corresponding to 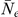 = 512 with 2σ range ±162 (pink region in Figure 3).

Figure 3 plots the likelihood versus *N_e_* of the scenarios considered above, under a neutral model. While we investigated a large range of possible *N_e_*'s (range 5–50,000), all the aspects of the data we have tested are not significantly unlikely under a neutral scenario within the *N_e_* range consistent with our other analyses (Table 1). In particular, all the likelihoods for these individual aspects of the data are larger than 0.05 for *N_e_*≈800. Thus, we cannot reject a neutral model for these data, even though some uncertainty may remain because of the low likelihood (p<0.1) of observing ≥3 taxa of subpopulation s5 at day 522. The modeling results were robust to whether potential recombinants were included or excluded (Figure S4).

These results were consistent with classical tests (Tajima's D, and Fu and Li's D* [19], [20], [21]) that showed no significant deviation from neutrality when the whole dataset was analyzed.

### Subpopulation frequency fluctuations may affect the observed evolutionary rate

Because the subpopulations were present at different frequencies over time we were interested in the potential impact of such fluctuations on the measured evolutionary rate. Clearly, the apparent substitution rate varied greatly over time (Figure S5). Thus, naively measuring the genetic difference between time points may mislead the estimation of the *de novo* substitution rate. However, the fluctuations may be another mechanism that HIV-1 uses to adapt and evolve its population structure. Hence, to accurately estimate the substitution rate one must take the phylogeny into account.

We used a Bayesian coalescent method to infer the *de novo* substitution rate within each subpopulation. To account for the frequency variation of each subpopulation we used the Bayesian skyline demographic model, which allows *N_e_* to vary over time in a non-parametric way. The evolutionary rate was inferred as a hyper-parameter using separate, independent trees to describe each of the subpopulations. A relaxed clock model was used to infer a hyper-parameter with individual distributions for each subpopulation. The mean estimated within-subpopulation substitution rate was 2.33×10^−3^ substitutions site^−1^ year^−1^, with a 95% highest posterior density (HPD) interval of 0.94–3.74×10^−3^ substitutions site^−1^ year^−1^. In agreement with our frequency analysis above (Figure 2), no significant deviation from a constant *N_e_* could be observed as the Bayesian skyline could contain a constant *N_e_* within the 95% HPD.

**Figure 4:**
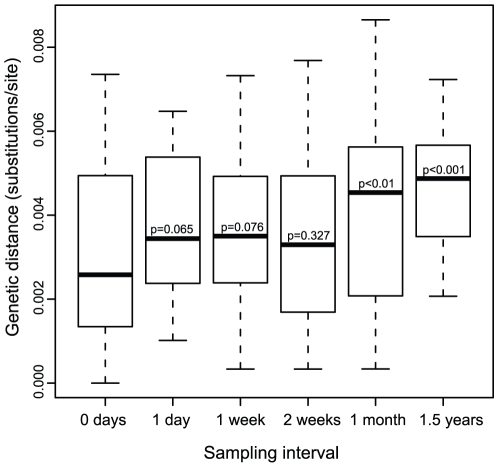
Genetic divergence in subpopulation s6. Mean pairwise distances were calculated between sequences sampled with different time intervals. At an interval of one month or more, the genetic distances were significantly greater than the intra-sample diversity (0 days interval) (p<0.01, Wilcoxon rank sum test). Sampling intervals of 1–2 days and 3–4 weeks were estimated together and are named 1 day, and 1 month, respectively.

The genetic divergence (nucleotide substitution rate) was further analyzed in subpopulation s6, which constituted the largest group of sequences (Figure 4). The mean pairwise distance (MPD) between clones sampled at the same time, i.e. the population diversity (0 days), was then compared to the divergence at later sampling times. Interestingly, we found that the MPD of sequences separated by about one month's interval (and longer) differed significantly from the intra-sample diversity (p<0.01, Wilcoxon rank sum test), but no significant divergence was seen in shorter time intervals. Hence, this HIV-1 subpopulation had moved significantly in sequence space after about a month.

## Discussion

HIV-1 evolves by introducing mutations (substitutions, indels, recombination) through a “sloppy” replication mechanism, mainly due to the unfaithful replication by the viral reverse transcriptase. These mutations are often deleterious [16] or otherwise detrimental to virus fitness [22], [23]. However, some mutants have an advantage as they may allow escape from immune surveillance [24], [25] or more effective infection of certain tissue compartments or cell types, such as cells in the brain or the genital tract [26], [27], [28] or naïve CD4+ T-cells, which express CXCR4 [29], [30]. Here we show that in addition to the mutational processes, HIV-1 can alter its population structure by frequency shifts among subpopulations. Because we analyzed a relatively small number of sequences per time point, we were careful to include the sampling into our analysis method. Over short time (days, weeks, months) these fluctuations were consistent with a constant population size, and most mutations that occurred at this time scale were neutral or only weakly selected. On longer time scales we noticed that the fluctuations became significant movements.

Here, we focused on short-term evolutionary processes (days, weeks, and months), whereas earlier studies as well as the generation of, and escape from, neutralizing antibody responses involve time frames of months to years [31], [32], [33], [34]. Clinically, our patient was classified as a slow disease progressor. Genetically, the virus population in our patient was described by co-existing subpopulations. Thus, it is interesting to compare the HIV population genetics of our patient to previously published patients with normal and slow disease progression. In a study by Shankarappa et al, 5 patients had slow disease progression (p2, p3, p7, p9, p11) and 5 had normal progression (p1, p5, p6, p8) [2]. These patients were followed over many years, but interestingly over a sampling period equivalent to ours (522 days, but with fewer samples), patients in both clinical groups showed subpopulation structure qualitatively similar to our patient (Figure S6). Thus, the short-term evolution we study here is likely representative for many patients regardless of disease progression rate.

One might have expected that the persisting subpopulations found in this patient were controlled by balancing selection [35], [36], [37]. Directional selection would have favored the fittest of the subpopulations and it would have been unexpected to see them coexist for so long, let alone to have several well separated subpopulations, which implies that they have existed for longer than the study period. Hence, some type of frequency-dependent selection, where the fitness of a variant/subpopulation is dependent on its relative frequency, would be the alternative hypothesis to neutral drift. Here we show that although the immune system partly controls virus replication during the chronic phase of the disease, particularly well in a slow progressor, and where one would expect escape mutants to dominate in *env*, the genetic evolution is consistent with a neutral process, at least over the time period studied here. In agreement with this, it was recently shown that genetic drift was a main contributor to HIV evolution in culture [38]. Similarly, in several other virus systems with large population sizes and high mutation rates, where deterministic processes are expected, genetic drift was shown to have a larger effect than expected [39], [40], [41], [42]. Furthermore, stochastic evolution during drug treatment of HIV-1 has previously been demonstrated [11]. Thus, also *in vivo* evolution of HIV-1 during the chronic phase may be largely described by neutral and stochastic processes. We speculate that this might be due to that the immune system “hits” all subpopulations with near equal efficiency.

Our tests for neutrality of the subpopulation frequency fluctuations are of necessity informal. A more formal procedure would assess the likelihood of the data under neutral models with varying *N_e_* and compare with models that additionally include either balancing or directional selection. There exist methods for estimating *N_e_* from multi-allele temporal data (e.g. [32]), as well as methods for inferring directional selection from two-allele temporal data (e.g. [33]). However, we are not aware of likelihood methods that include balancing selection and multiple alleles, and their development is beyond the scope of the present study. Hence, we have relied on a less formal method that may not have optimal power, but nevertheless is informative. In addition, our *N_e_* estimate from sequence data were in the order of previously estimated *N_e_* of HIV-1 in chronic infection [8], [13], [43], however, subpopulation structure or non-neutral evolution may bias these estimates, therefore we included a large range of plausible *N_e_* in our test of neutrality (*N_e_* = 5–50,000).

We have sampled free HIV-1 viral particles in plasma but we do not know where these virions were produced. The degree of compartmentalization of HIV-1 replication is uncertain; some researchers have found evidence of compartmentalization whereas others have not [29], [44], [45], [46], [47], [48], [49]. However, in untreated patients most virus in plasma is produced by short-lived activated CD4+ T-lymphocytes [50], [51] and there is no or limited compartmentalization between virus in plasma and lymphocytes [44], [52], [53]. Thus, the plasma virus population should be competing for the same resources, which would justify our analysis of whether balancing selection exists. However, we cannot exclude that the frequency fluctuations we see may be due to differential production from different compartments. The subpopulations were present in actively replicating virus since 1) the subpopulations were detected over time at high frequencies (i.e., detected in 7–11 single molecules per time point), 2) the molecules sequenced must represent virions which were replication competent at least in the previous generation, and 3) subpopulation s6 evolved at a measurable evolutionary rate.

It was interesting to note that PNGS were significantly over-represented among positively selected sites. Glycosylation and movement of glycans have been suggested to be an important immune escape mechanism of HIV-1 [31], [32], [34], [54]. Our data are compatible with such a scenario, which suggests that immune escape and positive selection on PNGS may have contributed to the evolution of the genetic subpopulations in our patient. Previously, it was demonstrated quantitatively that a wide range in strength of the autologous neutralizing antibody response between patients and corresponding differences in the impact on the viral population [34]. The fact that we observed positive selection on PNGS, and earlier studies have shown consecutive replacement HIV-1 *env* sequences [2], [55] and continuous neutralization escape [33], [34], do not contradict our observation that evolution in our patient overall was consistent with a neutral model of evolution.

The estimation of the evolutionary rate may be misled if phylogenetic relationships are not accounted for. This may occur in a naïve analysis where genetic distances between two (or more) time points are directly compared. Without accounting for the phylogeny, especially when the population is divided into clear subpopulations, frequency shifts between existing variants will masquerade as *de novo* mutations. We show that in *env*, which has the highest evolutionary rate in the HIV-1 genome, the number of *de novo* mutations accumulated a significant distance after about one month within a subpopulation. Hence, this suggests that sampling more frequently than this may not be useful to estimate the evolutionary rate in patients during the chronic phase. This is in good agreement with previous estimates of significant temporal changes at 22 months based on genomic sequences of similar length (∼1100 nt) in *gag-pol* [13], which evolves much slower than *env*. Note, however, that selection during drug treatment may potentially act upon existing variants/subpopulations at a much faster rate [56], [57], but, as we show here, during chronic, asymptomatic, untreated viral infection evolution proceeds mostly by neutral drift over shorter time frames.

In conclusion, we have performed high-frequency sampling of HIV-1 evolution in a chronically infected, untreated patient with slowly progressing disease. We shown that multiple well-separated subpopulations may persist for years, and over weeks and possibly months their frequencies remained constant. Over the time period of years, however, their fluctuations became significant, but were still consistent with a neutral model of evolution. However, sequence-based methods showed that individual sites had experienced positive selection, possibly as the subpopulations were being formed over several years. While the subpopulation frequencies fluctuated consistent with neutrality, the divergence within a subpopulation showed a temporal trend that was resolved at about one month's time.

## Materials and Methods

### Patient and samples

The patient was a treatment naive, asymptomatic man that had been HIV-1 infected for approximately 7 years at the start of the study (day 1). The plasma viral load had been stable and relatively low for several years and ranged from 450 to 1220 copies per ml during the main study period. The CD4 count was around 600 at the time point for the first sample, but with previous CD4 fluctuations including values below 500. Thus, the patient did not fulfill the definition of a long-term non-progressor (CD4 counts >500 for more than ten years without antiretroviral), and instead we classify the patient as a slow progressor [58]. In support of this, the patient was put on treatment after more than 15 years of infection.

Blood samples were collected each morning for 12 consecutive days (day 1 through day 12) and then once every week for 3 weeks (day 18, 25 and 32). One later sample, that was collected 1.5 years after the first sample (day 522) was also analyzed as well as an earlier sample that was collected 1.5 years before the start of the study (day −664). Plasma was prepared by centrifugation at 2000 rpm for 10 min at room temperature and stored at −70°C until analysis. Viral RNA was extracted from plasma using the Nuclisense RNA extraction kit (NASBA Diagnostics, Organon Teknika, Boxtel, The Netherlands) according to the manufacturer's instructions, and cDNA synthesis was performed using the First-Strand cDNA synthesis kit (Amersham Pharmacia Biotech, Uppsala, Sweden).

### Ethics statement

The patient gave written consent and the study was approved by the regional ethics committee (Karolinska Sjukhuset, Lokal forskningsetikkommitte Nord) in Stockholm (Dnr: 98–336).

### Amplification, cloning and sequencing

Single viral molecules were obtained by limiting dilution of the cDNA [14]. The method was selected to minimize the influence of PCR errors in the sequences and to allow sequencing of the entire *env* gene. According to the Poisson distribution, the likelihood that a positive PCR reaction originates from a single molecule is 0.95 if the fraction of positive reactions is 1∶3. After a dilution series, we determined the template load for each PCR and diluted our template accordingly. Hence, positive PCR samples from dilutions containing less than 1∶3 of positive reactions were sequenced and analyzed. The single molecule status was confirmed by screening for mixed nucleotide positions in the final sequence chromatograms and sequences with mixed positions were excluded. Hence, this procedure will identify PCR errors after the cDNA synthesis as they would be seen in the chromatograms at frequencies ≤25%. In addition, bidirectional sequencing was performed. In one sequence only one mixed position (at 50% in overlapping sequence fragments) was detected (A/G) and in this case both possible sequences were included. A 3.1-kb region covering *vpu*, *env*, and one-half of *nef* was amplified and sequenced as previously described [59]. A nested amplification was used with outer primers JL86 (5′-CCGTCTAGATGCTGTTTATTCATTTCAGAATTGG-3′) and JL89 (5′-TCCAGTCCCCCCTTTTCTTTTAAAAA-3′), and inner primers ED3 (5′-TTAGGCATCTCCTATGGCAGGAAGAAGCGG-3′) and JL88 (5′-TAAGTCATTGGTCTTAAAGGTACCTG-3′). The Expand Long Template kit (Boehringer Mannheim, Indianapolis, IN) was used according to the manufacturer's recommendations and a hot start was achieved by separating the primers and the template from the enzymes (Tgo DNA polymerase and Taq DNA polymerase) with a wax layer (DynaWax; Finnzymes, Espoo, Finland). The PCR program was 94°C for 10 sec, 55°C for 30 sec, and 68°C for 4 min for a total of 30 cycles. Concentrations of 0.4 µM primer and 0.2 mM total dNTP in a final volume of 50 µl were used, and 2 µl of first-round product was transferred to the second-round reaction. Positive reactions were purified using the GFX purification kit (Amersham Biosciences Corp, Piscataway, NJ) and directly sequenced with a walking primer approach using standard dideoxy-terminator fluorescent automated sequencing methodology (Applied Biosystems, Foster City, CA) on ABI 310 or 3100 sequencing machines. Sequencing primers were designed so that each nucleotide of the PCR fragment was detected by at least two separate primers. Hence, all nucleotide calls were made based on at least two sequencing reactions, ensuring high base-call accuracy. The sequences were evaluated and assembled into contigs using Sequencher software (Genecodes Inc, Ann Arbor, USA). The sequences are deposited in Genbank under the accession numbers: JN251812–JN251888.

### Sequence analyses

Sequences were manually aligned to HIV-1 reference sequences using the Se-Al software [60]. The program Modeltest v 3.7 [61] was used to search for the substitution model that best described the evolution of the dataset. ML trees were inferred using PhyML 3.0 [62] using 5 random starting trees with SPR and NNI tree search algorithms. Substitution model parameters were estimated from the data. Topological uncertainty was estimated using maximum likelihood evaluated non-parametric bootstrap analysis with 1000 replicates. Whether the sequence data generally supported a neutral model of evolution was tested using Tajima's D-test, Fu and Li's D*-test and Fay and Wu's H-test [19], [20], [21]. Coalescent estimation of N_e_ was made with the coalescent-likelihood programs Recombine and Fluctuate implemented in the Lamarc 2.1.3 package [63] as well as by calculating the mean-pairwise distance (MPD) using the program PAUP* [64].

To exclude possible laboratory contamination and sample mix-up, a phylogenetic tree was constructed where other subtype B *env* sequences from the HIV sequence database [65] were included together with the current dataset. This analysis showed that all our sequences formed a monophyletic cluster (not shown).

### Recombination analysis

In order to assess the extent of recombination in our dataset, and possibly identify the recombinants, we applied a procedure that has been shown to be able to identify intra-host recombination [66]. Conflicting phylogenetic signals in the dataset are visualized using the Neighbor Net (NNet) algorithm [67] implemented in SplitsTree version 4.10 [68] and the presence for recombination signal is then specifically tested with the pairwise homoplasy index (PHI) statistic [69]. The PHI statistic measures the similarity between closely linked sites and the significance of the observed test statistic is obtained using a permutation test. If there is no recombination in the data the genealogical correlation of adjacent sites is invariant to permutation [70]. But in the presence of finite recombination, the order of the sites is important, and distant sites will tend to have less genealogical correlation than adjacent sites. [69], [70] Subpopulations were screened one at a time by the PHI-NNet test. Intra-subpopulation recombinants were removed before screening for putative inter-subpopulation recombinants. As the identification process of putative recombinants may be subjective we wanted to control for human bias in selecting putative recombinants. We therefore randomly removed an equal number of sequences as were determined recombinant and calculated the PHI p-value. This randomized reduction was performed a hundred times.

To verify that the removal of the putative recombinants as determined by the PHI-NNET analysis rendered the dataset free from recombination signal we tested the two alternative datasets with the single breakpoint analysis available at www.datamonkey.org/GARD/ [71].

### Lineage- and site-specific selection analysis

Recombinant sequences, as determined by the PHI-NNet test, were removed and the alignment stripped so that only single-frame coding regions were present, i.e., only *env* without *vpu/rev*. A few spurious stop-codons were conservatively changed to the weighted nucleotide in the corresponding column of the alignment. This will reduce diversity and will not lead to false positive selection detection. We tested whether the identified subpopulations had evolved under different selection pressures by using GAbranch [72] available at the www.datamonkey.org website [73]. GAbranch automatically partitions all branches in the tree into several selective regimes and performs multi-model inference enabling us to infer dN/dS rates for each branch in the tree without subjectively choosing which branches to test for differential selection. In addition, we tested the subpopulations for site-specific selection or variation using Nielsen & Yang's hierarchical model-pairs (M0, M1a, M2a, M3,) in HyPhy [6], [74]. Individual amino acid changes were identified over the ML tree within or between subpopulations using MacClade [75].

### HIV-1 population subdivision

Putative subpopulations were identified by high bootstrap values as above. To test whether these subpopulations were statistically significant, we conducted a test for population subdivision originally developed by Hudson et al. and further developed to test HIV-1 intra-patient evolution by Achaz et al. [13], [70]. The method calculates matrices of pairwise sequence differences for the putative subpopulations as well as for the whole dataset. To assess the significance of the structure the sequences are randomly relabeled into new subsets of populations (keeping n_1_ and n_2_ constant), which generates a p-value for the probability that the structure observed was due simply to chance. The test does not rely upon a common genealogy for all sites, which makes it robust to the presence of recombination [13].

### Significance of subpopulation frequency fluctuations

Our significance test includes stochastic effects due to limited sample sizes. To determine whether subpopulation frequencies were significantly fluctuating within the patient, we asked whether the sample frequencies observed on each day *J* were consistent with the within-patient frequencies inferred from days 1…*J*-1. On each day *J* we have *N_J_* total observed sequences, within which subpopulation *i* has frequency count *f_iJ_*. If we assume the within-patient frequencies *φ_i_* are constant, then given the observations from days 1…*J*-1 the maximum-likelihood estimate 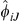 of the within-patient frequency of subpopulation *i* is simply the fraction of all previously observed sequences that are from subpopulation *i*. To assess whether the observed 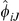 frequencies *f_iJ_* on a given day *J* are consistent with the inferred patient frequencies, we use Pearson's χ^2^ statistic:
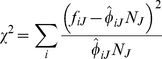
(1)The χ^2^ statistic sums over all subpopulations *i* the deviation between the observed counts *f_iJ_* and the expected counts 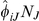.

In assessing the significance of the observed χ^2^ values, we account for our uncertainty in estimating the within-patient frequencies from the day 1…*J*-1 data using simulations of the estimation process. To do so, we begin with our maximum-likelihood estimate 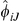 of the within-patient frequencies from the observed data. We then generate simulated data sets using constant within-patient frequencies of 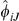, matching the number of samples *N_J_* from each day. For each of these simulations, we calculate χ^2^ for day *J* using population frequencies estimated from simulated days 1…*J*-1. One subtlety is that χ^2^ will be divergence if a subpopulation is first observed on day *J*. To avoid this, when calculating χ^2^ for both the real and simulated data, we introduce one addition count (a pseudocount) for each subpopulation on the first day. Our p-values are then the proportion of simulated data sets that yield a larger χ^2^ than the real data.

### Simulation and likelihoods of neutral subpopulation fluctuations

Our neutral simulations begin at day 1 with a population of *N_e_* individuals, divided amongst the 5 subpopulations based on their inferred within-population frequencies 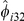 from days 1…32. In each generation a new population of *N_e_* individuals is created by sampling with replacement from the prior generation, i.e., a Wright-Fisher model of reproduction. The generation time for HIV-1 is assumed to be 2 days [9], so that 260 generations separate our samples at day 1 and day 522. For each simulation, based on the final subpopulation frequencies after 260 generations, we calculate the likelihood of observing particular aspects of the real day 522 data. The likelihood of sampling a given number of sequences from population *i* can be calculated directly from the multinomial probabilities. The likelihood of observing *n* subpopulations out of *T* possible is calculated by summing the likelihood of obtaining 0 observations for *T-n* subpopulations, carefully accounting for the number of ways to do this. Our overall likelihoods represent the average over 10^4^ population simulations. Also, our results are unchanged if we incorporate our uncertainty in estimating 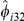 by initializing each simulation with different within-patient frequencies consistent with our observations from days 1…32.

### Evolutionary Rate Estimations

The programs TreeRate [76], [77] and BEAST v1.5.1 [78] were used to infer evolutionary rates. TreeRate optimizes the root and the evolutionary rate for a given tree by minimizing tip-height variances at two specified sampling times. The given tree was inferred by PhyML 3.0 as described above, thereby not preconditioned on a molecular clock. In addition we used Bayesian analysis (BEAST) assuming a relaxed molecular clock (uncorrelated lognormal) and a non-parametric population growth model (Bayesian skyline).

## Supporting Information

Figure S1 **Neighbor-Net diagrams showing the evolutionary relationships in the viral population including incompatible signals.** Clones from the different time points are indicated with different symbols and colors as shown. The subpopulations are labeled with letters s1–s6 **A**. All 77 taxa with 8 putative recombinants as determined by the PHI test and indicated with a star. **B**. The resulting network when these 8 putative recombinants were removed.
(EPS)

Figure S2 **Cladogram of the non-recombinant sequences where the dN/dS values inferred through GAbranch are shown.** Taxa labels are colored according to genetic subpopulation.
(EPS)

Figure S3 **Correlation of the normalized frequency of amino acid (aa) substitutions (A) and normalized potential N-linked glycosylation site (PNGS) replacements (B) to the probability of strong positive selection pressure.** The frequencies of aa and PNGS replacements were normalized by the number of tree branches between subpopulations (*N* = 8) or within subpopulations (*N* = 130). The selection pressures were partitioned into 3 rate classes (dN/dS = 3.92, dN/dS = 0.55, dN/dS = 0.13), optimized using the Nielsen-Yang model M3 in HyPhy [6], [74]. The probability of each site of belonging to the dN/dS = 3.92 class was used to measure the strong positive selection pressure. The correlations to strong positive selection were R = 0.78 (between subpopulation aa substitutions), R = 0.69 (within subpopulation aa substitutions), R = 0.40 (between subpopulation PNGS replacements), and R = 0.38 (within subpopulation PNGS replacements). The response to strong positive selection, as measured by OLS regression slopes, was 23 times stronger to between than within subpopulation aa substitutions and 25 times stronger to between than within subpopulation PNGS replacements (p<<0.001, F-statistic, in both cases).
(PDF)

Figure S4 **Likelihood under a neutral model of aspects of data with putative recombinants excluded.**
(EPS)

Figure S5 **The evolutionary rates given in percent substitutions per site and year, measured between all time points with TreeRate.** Each arrow begins at the first time-point, and the end of each shaded area represents the second time point. The impact of inclusion or exclusion of putative recombinant is shown; the upper part of each arrow represents exclusion of recombinants (Figure S1), and lower part of arrow represents the results when recombinants were included.
(EPS)

Figure S6 **Genetic diversity and divergence over time in our patient and previously published patient data.** Our patient (Study patient) was sampled over approximately 3 years, with most samples days, weeks and months apart up to 522 days (∼1.5 years). Comparing our results to an equivalent sampling period of patients in a study by Shankarappa et al [2], shows that regardless of disease progression rate similar subpopulation structure as in the study patient occurs in at least 5/9 Shankarappa patients (3, 5, 7, 8, 9). Shankarappa patients 2, 3, 7, 9, 11 had slow disease progression, as our patient, and the others normal disease progression. All trees are on the same scale (see scale bar) and the sampling time intervals are also on the same scale, 120 evenly divided colors over 12 years (12 colors shown in legend).
(JPG)

Table S1 **Subpopulation frequency fluctuations.** Excluding recombinant sequences from our analysis, we obtain the results shown in Table S1 for the significance of within-patient frequency fluctuations.
(DOCX)

Table S2 **Subpopulation frequency fluctuations are consistent with neutral drift.** Table S2 shows, excluding recombinants, the subpopulation frequencies inferred from our data, along with the expected and observed counts in day 522. Again, we observe several aspects of the data that are informative about potential deviations from neutrality. We test for the likelihood of 1) s1 not being observed, 2) s3 being observed, 3) s4 not being observed, 4) s5 being observed at frequency 2 or greater, 5) s6 being observed at frequency between 3 and 5 (indicating a fluctuation of less than 1 from expected), and 6) observing 3 or more populations. Figure S1 shows the results that none of these aspects of the data are significantly unlikely (p<0.05) under a neutral model.
(DOCX)
