# Supplementary figures and images for: Daily Sampling of an HIV-1 Patient with Slowly Progressing Disease Displays Persistence of Multiple env Subpopulations Consistent with Neutrality
Source: PLoS One. 2011 Aug 2;6(8):e21747. doi: 10.1371/journal.pone.0021747 (PMC3149046; doi:10.1371/journal.pone.0021747)

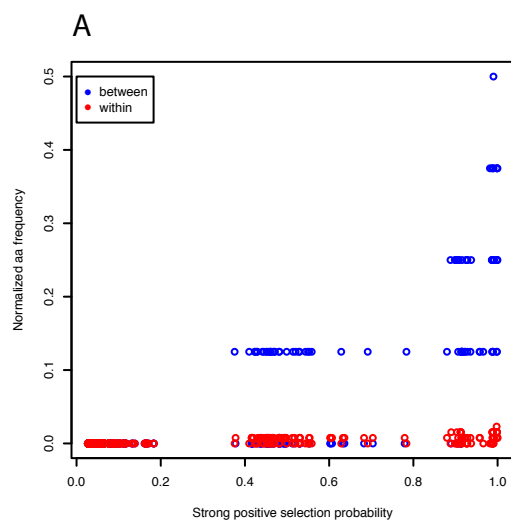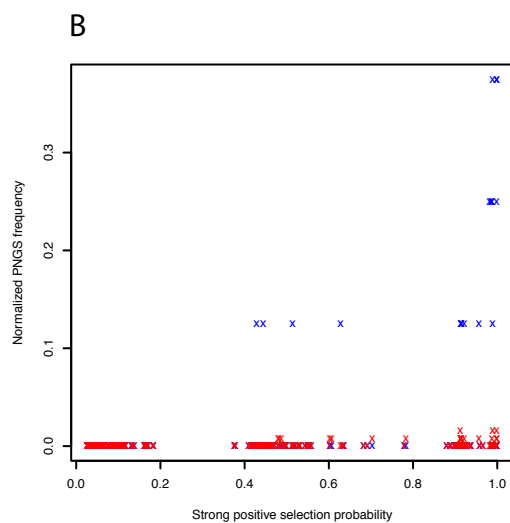

Supplement: Figure S3 — Correlation of the normalized frequency of amino acid (aa) substitutions (A) and normalized potential N-linked glycosylation site (PNGS) replacements (B) to the probability of strong positive selection pressure. The frequencies of aa and PNGS replacements were normalized by the number of tree branches between subpopulations (N = 8) or within subpopulations (N = 130). The selection pressures were partitioned into 3 rate classes (dN/dS = 3.92, dN/dS = 0.55, dN/dS = 0.13), optimized using the Nielsen-Yang model M3 in HyPhy [6], [74]. The probability of each site of belonging to the dN/dS = 3.92 class was used to measure the strong positive selection pressure. The correlations to strong positive selection were R = 0.78 (between subpopulation aa substitutions), R = 0.69 (within subpopulation aa substitutions), R = 0.40 (between subpopulation PNGS replacements), and R = 0.38 (within subpopulation PNGS replacements). The response to strong positive selection, as measured by OLS regression slopes, was 23 times stronger to between than within subpopulation aa substitutions and 25 times stronger to between than within subpopulation PNGS replacements (p<<0.001, F-statistic, in both cases). (PDF) [file pone.0021747.s003.pdf]

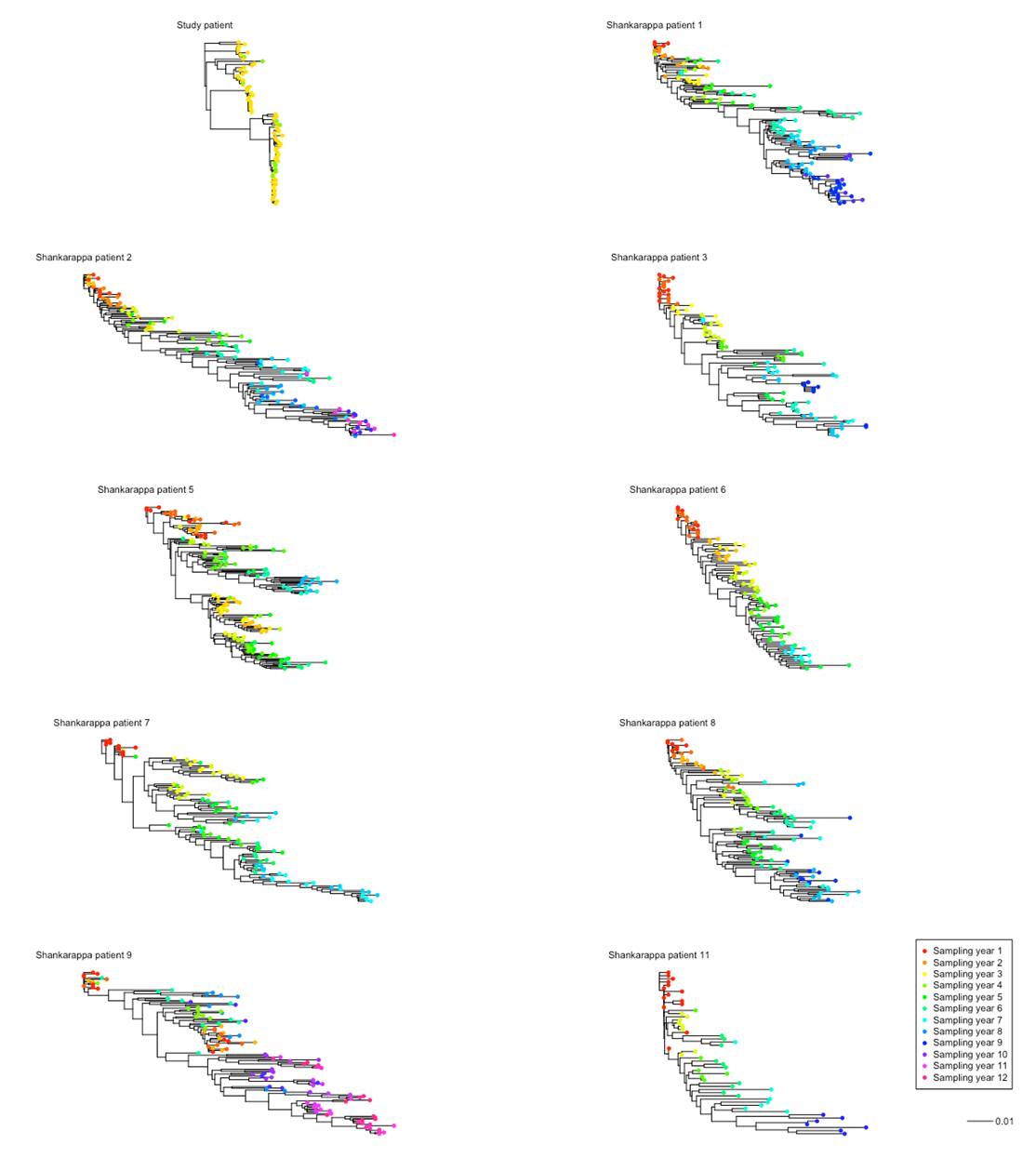

Supplement: Figure S6 — Genetic diversity and divergence over time in our patient and previously published patient data. Our patient (Study patient) was sampled over approximately 3 years, with most samples days, weeks and months apart up to 522 days (∼1.5 years). Comparing our results to an equivalent sampling period of patients in a study by Shankarappa et al [2], shows that regardless of disease progression rate similar subpopulation structure as in the study patient occurs in at least 5/9 Shankarappa patients (3, 5, 7, 8, 9). Shankarappa patients 2, 3, 7, 9, 11 had slow disease progression, as our patient, and the others normal disease progression. All trees are on the same scale (see scale bar) and the sampling time intervals are also on the same scale, 120 evenly divided colors over 12 years (12 colors shown in legend). (JPG) [file pone.0021747.s006.jpg]
