# Supplementary material for: Daily Sampling of an HIV-1 Patient with Slowly Progressing Disease Displays Persistence of Multiple env Subpopulations Consistent with Neutrality
Source: PLoS One. 2011 Aug 2;6(8):e21747. doi: 10.1371/journal.pone.0021747 (PMC3149046; doi:10.1371/journal.pone.0021747)
Supplement: Table S1 — Subpopulation frequency fluctuations. Excluding recombinant sequences from our analysis, we obtain the results shown in Table S1 for the significance of within-patient frequency fluctuations. (DOCX) [file pone.0021747.s007.docx]

**Table S1.** Tests of constant within-patient subpopulation frequencies without putative recombinant sequences

| Day 3 | Day 11 | Day 18 | Day 25 | Day 32 | Day 522 |
| --- | --- | --- | --- | --- | --- |
| 0.585 | 0.535 | 0.169 | 0.145 | 0.148 | 0.064 |
