# Supplementary material for: Daily Sampling of an HIV-1 Patient with Slowly Progressing Disease Displays Persistence of Multiple env Subpopulations Consistent with Neutrality
Source: PLoS One. 2011 Aug 2;6(8):e21747. doi: 10.1371/journal.pone.0021747 (PMC3149046; doi:10.1371/journal.pone.0021747)
Supplement: Table S2 — Subpopulation frequency fluctuations are consistent with neutral drift. Table S2 shows, excluding recombinants, the subpopulation frequencies inferred from our data, along with the expected and observed counts in day 522. Again, we observe several aspects of the data that are informative about potential deviations from neutrality. We test for the likelihood of 1) s1 not being observed, 2) s3 being observed, 3) s4 not being observed, 4) s5 being observed at frequency 2 or greater, 5) s6 being observed at frequency between 3 and 5 (indicating a fluctuation of less than 1 from expected), and 6) observing 3 or more populations. Figure S1 shows the results that none of these aspects of the data are significantly unlikely (p<0.05) under a neutral model. (DOCX) [file pone.0021747.s008.docx]

**Table S2.** Inferred, expected, and observed subpopulation frequencies without putative recombinant sequences

| Subpopulation | Inferred frequency from days 1… 32 | Expected counts on day 522 | Observed counts on day 522 |
| --- | --- | --- | --- |
| s1 | 0.213 +/- 0.105 | 1.7 | 0 |
| s2 | 0 | 0 | 0 |
| s3 | 0.082 +/- 0.070 | 0.66 | 1 |
| s4 | 0.148 +/- 0.091 | 1.18 | 0 |
| s5 | 0.032 +/- 0.046 | 0.26 | 2 |
| s6 | 0.524 +/- 0.128 | 4.2 | 5 |
